# Changes of EEG Spectra and Functional Connectivity during an Object-Location Memory Task in Alzheimer’s Disease

**DOI:** 10.3389/fnbeh.2017.00107

**Published:** 2017-05-31

**Authors:** Yuliang Han, Kai Wang, Jianjun Jia, Weiping Wu

**Affiliations:** ^1^Department of Neurology, Chinese PLA General HospitalBeijing, China; ^2^Department of Neurology, Chinese PLA 305 HospitalBeijing, China

**Keywords:** Alzheimer’s disease, object-location memory, electroencephalogram, power spectrum, functional connectivity, compensation mechanisms

## Abstract

Object-location memory is particularly fragile and specifically impaired in Alzheimer’s disease (AD) patients. Electroencephalogram (EEG) was utilized to objectively measure memory impairment for memory formation correlates of EEG oscillatory activities. We aimed to construct an object-location memory paradigm and explore EEG signs of it. Two groups of 20 probable mild AD patients and 19 healthy older adults were included in a cross-sectional analysis. All subjects took an object-location memory task. EEG recordings performed during object-location memory tasks were compared between the two groups in the two EEG parameters (spectral parameters and phase synchronization). The memory performance of AD patients was worse than that of healthy elderly adults The power of object-location memory of the AD group was significantly higher than the NC group (healthy elderly adults) in the alpha band in the encoding session, and alpha and theta bands in the retrieval session. The channels-pairs the phase lag index value of object-location memory in the AD group was clearly higher than the NC group in the delta, theta, and alpha bands in encoding sessions and delta and theta bands in retrieval sessions. The results provide support for the hypothesis that the AD patients may use compensation mechanisms to remember the items and episode.

## Introduction

Alzheimer’s disease (AD) is a degenerative brain disease, and episodic memory impairment is an early sign of it. Numerous studies have focused on the early detection of episodic memory impairment to resolve the differential diagnosis, estimate the disease progression, and determine eligible treatment.

Spatial memory, remembering the place of objects in our environment, is a prominent aspect of episodic memory. It is fundamental to human survival in everyday life. A few studies have demonstrated that spatial memory of AD patients is impaired, such as route learning tasks and scene memory assessment (Cherrier et al., 2001; deIpolyi et al., 2007; Iaria et al., 2007; Bird et al., 2010; Moodley et al., 2015). It was reported that impairments of spatial memory are typical and early symptoms of AD (Parra et al., 2010; Pertzov et al., 2012, 2013).

Memory for object-location is one type of spatial information memory. A number of investigations revealed that object-location memory is particularly fragile (Parra et al., 2010; Pertzov et al., 2012) and specifically impaired in patients whose damage area focused on medial temporal lobes (MTLs) (Pertzov et al., 2013, 2015). Thus, memory for object-location needs to be better researched in AD.

A few spatial memory paradigms have been used to study object-location memory in AD. The common object-location memory test consists of two procedures, including showing participants objects and performing recognition memory tests with time delay. For instance, patients were asked to pick out the items they have seen before and relocated them to previous positions (Kessels et al., 2000); the patients relocated the same objects to the positions previously presented (positions-only condition), different objects to the position marked by dots (object-to-position-assignment condition), different objects to the position without marks (combined condition) (Postma and De Haan, 1996; Kessels et al., 2002, 2010; van Asselen et al., 2008; Mazurek et al., 2015). Some paradigms also use pictures of real-life objects (e.g., buildings) on a map to recognize objects-location pairings (Kulzow et al., 2014; Edler et al., 2015). The 4 Mountains Test (4MT) is another brief behavioral test of spatial memory, and performance on it has been found to diagnose AD with high sensitivity and specificity (Hartley et al., 2007; Bird et al., 2010; Moodley et al., 2015). The primary design for 4MT paradigms encompassed spatial and non-spatial memory perceptions. The next two conditions involve alterations of light and vegetation color (Moodley et al., 2015). In this study, we used a new object-location memory task with less influence from other cognitive processing, in order to better understanding the spatial memory impairment in AD patients.

Electroencephalogram (EEG) has been used as the method to measure spatial memory. Numerous studies have found that brain oscillatory correlates of memory formation (Hanslmayr and Staudigl, 2014; Lee and Yang, 2014), and almost all frequency bands, from 3 Hz up to 100 Hz, are associated with the formation of memory (Nyhus and Curran, 2010; Hanslmayr et al., 2012; Hanslmayr and Staudigl, 2014). For example, activity in the theta and gamma frequency bands changes during the encoding and retrieval phases of a working-memory task, and the rate of correct responses correlated with the synchronization index. The analysis of electrophysiological data obtained while a spatial memory task is being performed may provide information about the function of neuronal systems involved in the type of spatial memory activity investigated. Theta band oscillatory in post-rhinal cortex and gamma band in CA3 of hippocampus were correlated with objects place memory in rats (Lu et al., 2011; Furtak et al., 2012). Delta, theta, gamma bands oscillations were correlated with spatial navigation in human (Snider et al., 2013; Park et al., 2014). P300, N200, N300 were the factors in an integrated object-location task in event-related potentials (ERPs) study (Simon-Thomas et al., 2003; van Hoogmoed et al., 2012).

Compared to the controls, AD patients showed an increase in slow (theta and delta) activities and a decrease in fast (alpha and beta) activities in resting-state EEG (Bennys et al., 2001), and decreased coherence at the alpha bands and beta bands (Locatelli et al., 1998; Leocani and Comi, 1999; Knott et al., 2000; Stam et al., 2003). The differences in spectral power and functional connectivity during cognitive tasks in relation to memory decline have also been studied (Rypma and D’Esposito, 1999; Hidasi et al., 2007; Bajo et al., 2010). Nonetheless, few oscillatory activity studies for object-location memory have been reported in AD patients.

Therefore, the aim of this study was to construct an object-location memory paradigm and explore objective signs of it, using parameters of electrophysiological EEG signal (spectral parameters and phase synchronization) in AD patients.

## Materials and Methods

### Subjects

The patient group included 20 mild AD patients, while the normal control (NC) group included 19 healthy older adults. The two groups were gender, age, and education matched (**Table 1**). All subjects were right-handed. The study was approved by the PLA general hospital Ethical Committee, and was performed in accordance with the Helsinki declaration. Written informed consent was obtained from all participants before study enrollment.

**Table 1:** Demographic data.

	NC	AD
Ages (years)	66.7 (6.7)	69.1 (8.8)
Education (years)	13.4 (3.2)	14.1 (2.0)
Gender (M/F)	7/12	8/12

*NC, normal control; AD, Alzheimer’s disease; M, male; F, female.*

Alzheimer disease patients fulfilled the criteria of probable AD dementia according to the National Institute on Aging-Alzheimer’s Association workgroups on diagnostic guidelines for AD (McKhann et al., 2011). Each patient was interviewed by expert neurologists and received a comprehensive medical assessment, including demographic data, past medical history, physical and neurological assessment, neuropsychological test, blood-screening tests, and structural magnetic resonance imaging (MRI) brain scan, to diagnose and exclude other causes of dementia.

All subjects took part in a battery of neuropsychological assessments, including Mini-Mental State Examination (MMSE) (Folstein et al., 1975), Clinical Dementia Rating (CDR) (Hughes et al., 1982; Morris, 1993), Neuropsychiatric Inventory Questionnaire (NPI) (Cummings et al., 1994), the Bristol Activities of Daily Living Scale (BADLS) (Bucks et al., 1996), Hachinski Ischemic Score (HIS) (Rosen et al., 1980), Rey-Osterrieth complex figure (ROCF) immediate recall and 10 min delay recall (Lezak, 2004; Strauss et al., 2006). The Taylor scoring unit method was used in scoring ROCF recall (Lezak, 2004). Patients diagnosed with mild AD in the patient group met the scores (20 ≤ MMSE ≤ 26, CDR = 1) in the neuropsychological test.

### Experimental Paradigm

The pictures used in this study were the same as the Snodgrass, some of which were selected by Chinese researchers to fit for Chinese subjects and were normative measured (Snodgrass and Vanderwart, 1980; Cycowicz et al., 1997). Those pictures were line drawings of common objects. From the total pool, 36 pictures were randomly selected. Pictures presented on the right or left were counterbalanced according to name agreement, familiarity, and visual complexity. E-prime software (version 2.0, Psychological Software Tools, Inc.) was used to present pictures and collect responses. All subjects were given the instructions prior to the test and there was a practice test consisting of two left pictures and two right pictures that the experimenter and subjects performed together in order to be sure that the subject completely understood the procedure. The practice pictures were different from those in the test phase.

**FIGURE 1:**
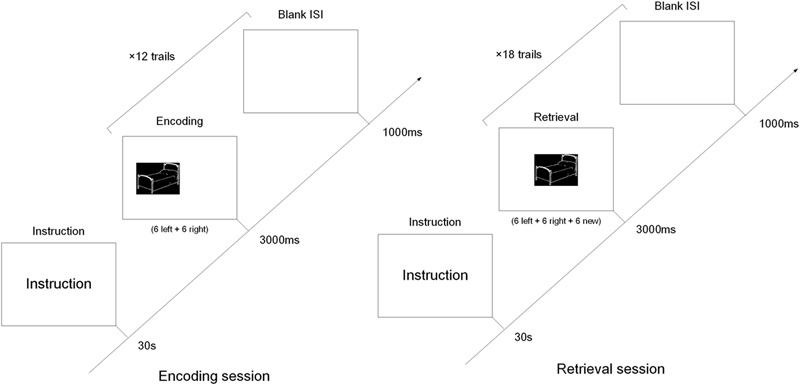
The diagram of the encoding and retrieval session of the object-location memory task. In the encoding session, six object pictures were presented left and another six object pictures right. In the retrieval session, the 12 pictures from the encoding session were randomly intermixed with six new pictures, and all pictures were presented in the middle of screen.

All presentations were on a 22-inch computer screen. The process was shown in **Figure 1**. The test started with the instructions displayed on the screen for 30 s, followed by a 1000 ms blank. Afterward, each session started with the presentation of a black square, similar in size to the test phase questions, for 3000 ms. In the encoding session, six pictures were randomly presented on the left and six on the right. Each picture was displayed for up to 3000 ms with a 1000 ms inter-stimulus-interval (ISI). While the pictures were being presented, subjects were instructed to press the “left” or “right” button on the keyboard to indicate the location of the picture. The duration of this screen was response terminated. The subjects were instructed to memorize the pictures for the subsequent memory test in the retrieval session.

The retrieval session was preceded by 30 s of rest following the encoding session. The 12 pictures in the encoding session were randomly intermixed with six new. All pictures were presented in the middle of the screen, and for the same length as in the encoding session. Subjects were instructed to press the “left” or “right” button when they recalled the location of pictures presented in the encoding session. If the picture wasn’t shown in the encoding session, they also pressed the “right” button (Cycowicz et al., 2001, 2008).

### EEG Recording

The EEG was recorded using 64 silver chloride electrodes mounted in an elastic cap using an ANT REFA-128 EEG measurement system (ANT Software BV, Enschede, The Netherlands). The electrodes were positioned according to an extended version of the international 10/20 system (Klem et al., 1999), and the electrode impedance was kept below 10 kΩ. Reference electrodes were placed on the mastoids. The vertical EOG (VEOG) was recorded from electrodes above and below the left eye. The EEG and EOG were recorded continuously at 512 Hz, high-pass filtered at 0.1 Hz and low-pass filtered at 100 Hz.

### Behavioral Performance

The trials with pictures located on the left in the encoding session were divided into two categories depending on the response given in the retrieval session: later remembered the location (LR-L) and later forgotten the location (LF-L). Trials in the retrieval session were classified as correctly recalled total (CR-T) [CR-T = correctly identified previously seen and recalled the location of pictures (HIT) + correctly rejected new stimuli (CR)] and false recalled total (FR-T) [FR-T = not recognized the location of old pictures (MISS) + new pictures incorrectly identified as old, i.e., false alarms (FAs)].

### EEG Data Analysis

#### Preprocessing

Electroencephalogram data preprocessing and analysis were performed using the Matlab R2012b software (MathWorks, Natick, MA, United States) and the EEGLAB software^1^. The average of the left and right mastoid was set as the reference electrode, and band-pass filtered from 0.1 and 45 Hz. Next, the EEG was segmented into epochs ranging from the stimulus mark appearing to the response mark appearing. Epochs and channels with visible artifacts were rejected when investigator visually inspected the EEG data. Eye movements artifacts were corrected using an independent component analysis (ICA) procedure. After visible artifacts correction, automated rejection of other EEG artifacts (e.g., muscles) was performed (criteria for rejection: >50.00 μV voltage step per sampling point, absolute voltage value > ±120.00 μV). All epochs with artifacts were excluded from the EEG analysis.

#### Power Estimation

In this study, EEG power was estimated in five sub-bands, including 1–4 Hz (delta), 4–8 Hz (theta), 8–13 Hz (alpha), 13–30 Hz (beta), and 30–45 Hz (gamma).

For each channel, the power spectrum density (PSD) was estimated by the Welch method with a Hamming window of 1 s and a 50% overlap. The power in sub-band [f1 f2] for some channel “ch” is calculated as follows:

$$P_{ch}(f_{1},f_{2})=\int_{f_{1}}^{f_{2}}psd_{ch}(f)df$$

Where the psd_ch_ is the PSD of the channel. The power for region R in sub-band [f1 f2] was eventually estimated by averaging the power in sub-band [f1 f2] of channels in the region R.

$$P_{R}(f_{1},f_{2})=\frac{\sum_{ch\in R}P_{ch}(f_{1},f_{2})}{N}$$

Where N is the number of channels in region R.

Finally, to evaluate which area showed a differential between AD patient and healthy controls, we statistically compared all channels across subjects using a non-parametric randomization test. A cluster-based randomization approach was used (Maris and Oostenveld, 2007). The method was described previously in Lange’s work (Lange et al., 2015), and the analysis was performed using FieldTrip toolbox (Oostenveld et al., 2011). The threshold of *t*-values was set at a value of *t* = 1.96 (i.e., *p* = 0.05). In cases where the *p*-value was smaller than an alpha-level of 0.05, we concluded that data in the two groups was significantly different.

#### PLI Calculation

Functional connectivity between different brain regions was computed using the phase lag index (PLI) (Stam et al., 2007). The segregation of the eight brain areas [left frontal (LF) (FP_1_, AF_7_, AF_3_, F_9_, F_7_, F_5_, F_3_, F_1_), left temporal (LT) (FC_5_, FT_7_, FT_9_, C_5_, T_7_, T_9_, CP_5_, TP_7_, TP_9_), left parietal (LP) (FC_3_, FC_1_, C_1_, C_3_, CP_1_, CP_3_), left occipital (LO) (P_1_, P_3_, P_5_, P_7_, P_9_, PO_3_, PO_7_, O_1_), right frontal (RF) (FP_2_, AF_8_, AF_4_, F_10_, F_8_, F_6_, F_4_, F_2_), right temporal (RT) (FC_6_, FT_8_, FT_10_, C_6_, T_8_, T_10_, CP_6_, TP_8_, TP_10_), right parietal (RP) (FC_4_, FC_2_, C_2_, C_4_, CP_2_, CP_4_), and right occipital (RO) (P_2_, P_4_, P_6_, P_8_, P_10_, PO_4_, PO_8_, O_2_)] is the same as that in the analytical system of neuromag (Elekta Oy, Helsinki, Finland).

The PLI is a measure that quantified consistent phase lead or lag between two signals. The method of PLI computed was described previously in Zeng’s work (Zeng et al., 2015). PLI can be computed from a time series of phase differences Δϕ(t_k_) (*k* = 1 … N) as follows:

$$\text{PLI=|}\langle \text{sign}[\Delta \;\phi (t_{k})]\rangle$$

The PLI ranges between 0 and 1. A PLI of zero indicates either no coupling or coupling with a phase difference centered around 0 mod π. And a PLI of 1 indicates perfect phase locking at a value of Δϕ difference from 0 mod π. The stronger the non-zero phase locking is, the larger the PLI will be.

Phase lag index was also computed in five sub-bands, including 1–4 Hz (delta), 4–8 Hz (theta), 8–13 Hz (alpha), 13–30 Hz (beta), and 30–45 Hz (gamma). The result of PLI for all pair-wise combinations of channels is an N × N matrix (N = 61, where each entry PLI, j is the value of PLI for the channels i and j) (Stam et al., 2007).

### Statistical Analysis

Student’s independent *t*-test was used to assess the difference of demographic data and behavioral performance data between two groups. Pearson’s *r* correlations were assessed between MMSE score and object-location task score in both groups. All statistical analysis were operated using IBM SPSS software (version 19.0).

## Results

### Behavioral Performance

The correct number and response time (RT) were calculated to compare the performance of the healthy older adults and the patients with mild AD. Details are shown in **Table 2**. MMSE scores (*t* = 10.219, *p* < 0.001), ROCF scores (immediate recall) (*t* = 5.391, *p* < 0.001) and ROCF scores (delayed recall) (*t* = 9.793, *p* < 0.001) of AD patients were lower than that of healthy older adults. More than half of the AD patients stated that they could not remember anything in ROCF delay recall test. The number of correctly recalled total (CR-T) was higher for the healthy older adults compared to the patients with mild AD (*t* = 4.192, *p* < 0.001). Furthermore, the number of later remembered the location was also more for the healthy older adults compared to the patients with mild AD (*t* = 8.722, *p* < 0.001). In the retrieval session, RTs for the CR-T of the NC group was significantly shorter than that of the AD group (*t* = -4.761, *p* < 0.001). MMSE correlated significantly with the number of LR-L items (*r* = 0.575, *p* < 0.01) and the number of CR-T items (*r* = 0.748, *p* < 0.01) in two groups. These results indicate that the function of object-location memory in AD patients is obviously impaired compared to healthy elders.

**Table 2:** Behavioral performance results.

	NC	AD
MMSE	29.2 (0.8)	23.6 (2.0)
ROCF score (immediate recall)	18.4 (6.3)	5.5 (5.1)
ROCF score (delayed recall)	19.2 (5.7)	2.2 (2.9)
LR-L accuracy (%)	75.8 (17.5)	47.5 (15.0)
CR-T accuracy (%)	85.8 (8.9)	58.3 (5.6)
CR-T response times (ms)	1241 (243)	1868 (446)

*NC, normal control; AD, Alzheimer’s disease; MMSE, Mini-Mental State Examination; ROCF, Rey-Osterrieth complex figure; LR-L, later remembered the location; CR-T, correctly recalled total. MMSE score of two groups was significantly different (*t* = 10.219, *p* < 0.001); ROCF score (immediate recall) of two groups was significantly different (*t* = 5.391, *p* < 0.001); ROCF score (delayed recall) of two groups was significantly different (*t* = 9.793, *p* < 0.001); LR-L accuracy of two groups was significantly different (*t* = 4.192, *p* < 0.001); CR-T accuracy of two groups was significantly different (*t* = 8.722, *p* < 0.001). In the retrieval session, RTs for the CR-T of two groups was significantly different (*t* = -4.761, *p* < 0.001). Correlations between MMSE score and LR-L (*r* = 0.575, *p* < 0.01); Correlations between MMSE score and CR-T (*r* = 0.748, *p* < 0.01).*

### Power Spectrum

**FIGURE 2:**
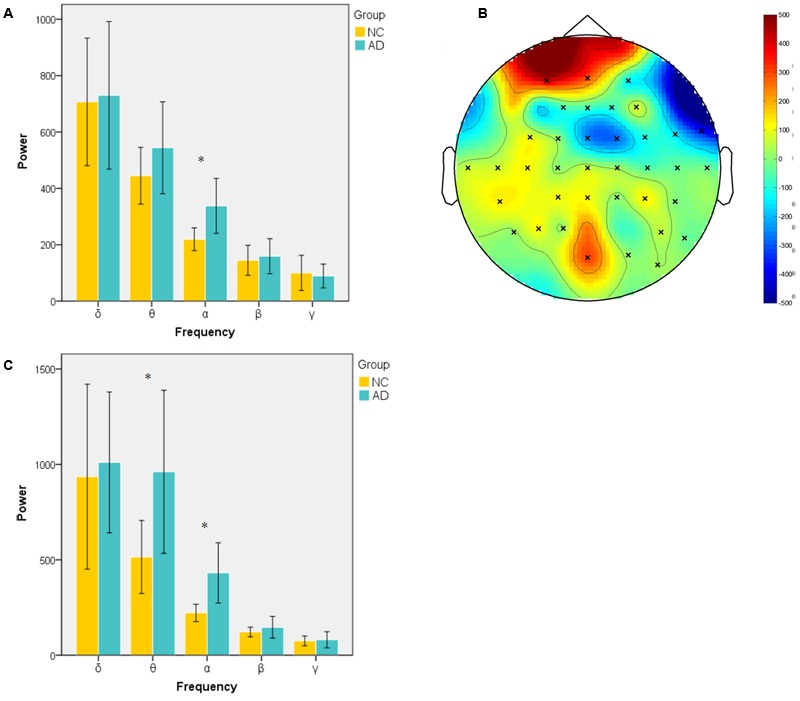
Spectral power in encoding and retrieval session. **(A)** Averaged power across all channels in encoding session for two groups at five frequency bands (delta, 1–4 Hz; theta, 4–8 Hz; alpha, 8–13 Hz; beta, 13–30 Hz; gamma, 30–45 Hz). Alpha band power of AD group was significantly higher than NC group; **(B)** topography of different areas between two groups at alpha band in encoding session (×: significantly different channels between two groups); **(C)** averaged power of two group in five frequency band in retrieval session. (^∗^*p* < 0.05).

Power during the successful encoding and retrieval stages was compared between the AD and NC groups. In our study, only object-location on the left and subsequent remembered (LR-L) items were analyzed because they reflect successful encoding object-location binding. The comparison was run across encoding and retrieval sessions in five frequency bands. The unpaired *t*-test was used to compare the average power of LR-L between two groups in every band frequency. In the encoding session, as shown in **Figure 2A**, the power of object-location memory encoding in the alpha band of the AD group was significantly higher than the NC group (*p* < 0.05). There were no significant differences in the delta, beta or gamma bands in the AD and NC groups. Further cluster analysis revealed that there were topographic differences in alpha power between two groups (**Figure 2B**). The difference of activation areas were almost distributed in the whole brain cortical areas. The AD group seems to have a trend toward frontal area activation increase (marked in red). Channels in the right hemisphere seem to show more channels with higher alpha power than the left hemisphere. In the retrieval session, as shown in **Figure 2C**, the power of object-location memory encoding in the alpha and theta bands of the AD group was significantly higher than the NC group (*p* < 0.05), while no significant differences in the delta, beta, or gamma bands between the AD and NC groups were observed. Finally, only the average power of whole channels was significantly different, while there were no topographic differences found between the two groups.

### Functional Connectivity

**FIGURE 3:**
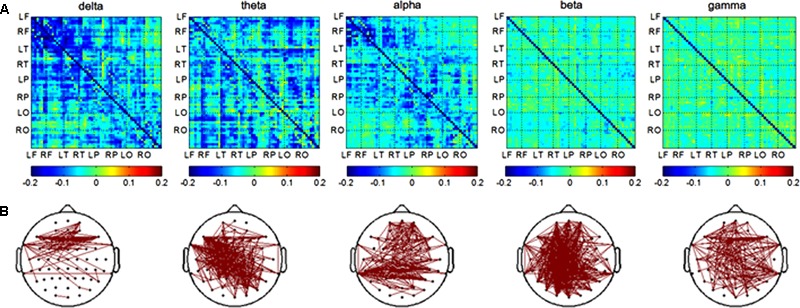
Functional connectivity in encoding session. **(A)** Difference of PLI value between two groups among eight brain areas (LF, RF, LT, RT, LP, RP, LO, RO) in five frequency band. NC < AD; **(B)** the PLI value of channels-pairs that had significantly difference between two groups in five frequency band.

In the encoding session, differences of channel pairs PLI value between the AD and NC groups among 8 brain areas were plotted in **Figure 3A**. The PLI value was higher in the AD group compared to that in the NC group. The PLI value of channel pairs that showed the significant difference between two groups is shown in **Figure 3B**. The number of different channels-pairs were 75 (delta), 267 (theta), 243 (alpha), 482 (beta), and 183 (gamma). Combining the results of **Figures 3A,B**, the most obvious differences in functional connectivity between different brain regions were in both hemispheres of the frontal and parietal regions in the alpha frequency band; frontal and temporal of both hemispheres in the delta band; and almost the majority of brain regions in the theta band.

**FIGURE 4:**
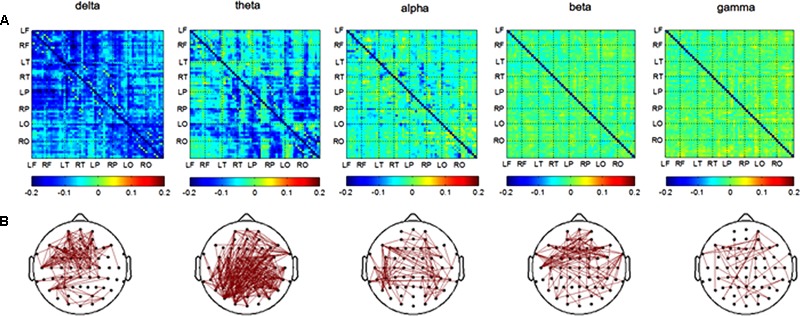
Functional connectivity in retrieval session. **(A)** Difference of PLI value between two groups among eight brain areas (LF, RF, LT, RT, LP, RP, LO, RO) in five frequency band. NC < AD; **(B)** the PLI value of channels-pairs that had significantly difference between two groups in five frequency band.

In the retrieval session, differences in channel pairs PLI value between the AD and NC groups among 8 brain areas were plotted in **Figure 4A**. The PLI value was higher in the AD group compared to that in the NC group. The PLI value of channel pairs had significant differences between groups (**Figure 4B**). The number of different channel pairs were 80 (delta), 353 (theta), 109 (alpha), 112 (beta), and 46 (gamma). Combined, the results of **Figures 4A,B** show that the most obvious differences in functional connectivity between different brain regions were in the parietal, temporal and occipital regions of both hemispheres in the theta frequency band, and in the frontal and temporal of both hemispheres in the delta band.

## Discussion

In our study, higher spectral power was found in alpha band frequencies in the AD group than in the healthy control group during object-location memory encoding. Furthermore, higher spectral power in the theta and alpha band frequencies was also found during memory retrieval. We also found that strengths of connectivity were obviously increased in the AD group when compared to the healthy control group prominent in delta, theta, and alpha band frequencies in memory encoding and delta and theta band frequencies in memory retrieval. The most obvious difference in functional connectivity during memory encoding was between the frontal and parietal in the alpha band and frontal and temporal in the delta band. During memory retrieval, the most obvious difference in functional connectivity was between the parietal, temporal, and occipital regions in the theta frequency band and between the frontal and temporal in the delta band. The significant differences between AD patients and healthy elders in our object-location memory task were in slow EEG oscillations. One possibility would be that slow oscillations modulate the fast at a higher level, which determine the general mode of processing (Knyazev, 2012).

### Encoding Stage of Object-Location Memory

At the encoding stage, the strength of functional connectivity in the theta band of AD patients was higher than that of healthy elders, which may indicate that cognitive function correlated with theta oscillations was impaired in AD patients. Theta rhythms are considered to the signs of taking and encoding new information (Colgin, 2013). Theta oscillations demonstrated the process in hippocampo-cortical feedback loops (Klimesch, 1999, 2012). The crucial role of theta rhythms in memory and spatial and temporal organization is well-investigated (Buzsaki, 2002). Studies in rats have demonstrated increase of mPFC-MTL theta phase in spatial memory tasks (Benchenane et al., 2010; Euston et al., 2012). In a study involving the hippocampus and the mPFC, the proportion of mPFC neurons that were phase-locked to hippocampal theta oscillations increased after successful learning of an object-place association (Kim et al., 2011). Since our study indicated a higher strength of functional connectivity in the theta band for AD patients compared to healthy elders, we hypothesize that the AD patients may use neural compensation to encode object spatial information.

The neural compensation demonstrated that brain actively attempts to recruit alternative structures which are not normally used to compensate for brain damage (Stern, 2009, 2012, 2013; Risacher and Saykin, 2013). Thus, brain size is not linearly correlated with brain function. Several studies have found that the neurodegenerative process of abnormal cortical oscillations in AD is accompanied by synaptic compensation mechanisms that are regarded to play a role in preventing the catastrophic amnesia associated with synaptic loss and maintaining excitement of neural circuits (Small, 2004; Turrigiano, 2011, 2012; Abuhassan et al., 2014).

A series of studies explored the concept of neural compensation either in elders, mild cognitive impairment (MCI), or AD patients (Rypma and D’Esposito, 1999; Cabeza et al., 2002; Zarahn et al., 2007; Steffener et al., 2009; Bajo et al., 2010). Deactivating local compensation will lead to rapid decline (cognitive deficit) of network dynamics in the theta and alpha bands (Moretti, 2015). Previous functional neuroimaging and physiological studies have reported several compensatory features such as the increase of activity in the MTL, the plasticity of the cholinergic system and regional cerebral blood flow (DeKosky et al., 2002; Dickerson et al., 2005; Dai et al., 2009). The method of event related desynchronization/synchronization (ERD/ERS), using auditory verbal memory task, was also indicated as a compensatory mechanism in MCI and AD (Karrasch et al., 2006).

In our study, the alpha band frequency power and strengths of functional connectivity of AD patients is higher during the encoding stage of object-location memory task compared to healthy elders. Alpha oscillations relate to a lot of cognitive domains such as perception, encoding, and recognition, which are guided by attention (Friston, 2009; Summerfield and Egner, 2009). Those processes are closely related to access of information in the knowledge system, which comprise of traditional long-term memory, procedural and implicit-perceptual knowledge (Klimesch, 1999, 2012). In addition, alpha activity plays an important role in attention by supporting processes within the attentional focus and inhibiting task-irrelevant memory entries (Sauseng et al., 2010; Benedek et al., 2014), and also correlation with maintenance of sensory representations (VanRullen and Macdonald, 2012). Brain activities during encoding and retrieval memory tasks involve a number of processes, including increased attention, use of elaborative strategies, and the formation of item-to-context associations. In order to complete the specific task in our study, all subjects need to combine their spatial attention and object-based attention, bind object to location, and maintain the information in their minds. Compared with the healthy elderly subjects, AD patients had to use other compensatory mechanisms for those process, thus having a higher alpha band spectral power.

In encoding sessions, the strength of functional connectivity in the delta band of AD patients was higher than that of healthy elders, indicating that the function correlated with delta oscillations is impaired in AD patients as well. While the role of delta frequency oscillations is still being debated, mounting evidence indicates that delta-band oscillations are mostly associated with old evolutionary basic motivational processes (Knyazev, 2012). Several studies considered delta as a ‘cognitive’ rhythm, such as Knyazev suggested that the motivational relevance of the task and the salience of the target stimulus were involved in enhanced delta activity (Knyazev, 2007). The cortical delta oscillations has been found as a mechanism in selective attention to rhythmic auditory or visual stimulus streams (Lakatos et al., 2008; Knyazev, 2012; Harmony, 2013). In our paradigm, in order to encode the item and bind location context successfully, subjects might pay attention to screening of picture representation and be involved in stimuli in search of motivationally salient cues that benefit for memory, which processing may reflect by delta oscillations. Therefore, our results may suggest AD patients recruit more neural network for motivation and attention.

### Retrieving Stage of Object-Location Memory

In our study, the higher spectral power and strengths of functional connectivity in theta band were found in AD patients while retrieving the object-location information. The most prominent differences in functional connectivity between different brain regions were between the parietal, temporal, and occipital of the two hemispheres. As previously mentioned, in the encoding stage, theta rhythm is associated with mnemonic function. Previous studies have also proved that recollection of contextual information is associated with increased theta power and phase synchronization (Guderian and Duzel, 2005; Fuentemilla et al., 2014). The functional connectivity between segregated brain regions in the theta frequency is also crucial to memory recall (Klimesch et al., 2010; Colgin, 2013). This indicated that AD patients might recruit compensatory resources for the hippcampo-cortical network.

For the hippocampus is crucial for recollection episode of location, cortical theta oscillations links hippocampal functioning for recollection. During recollection episode of location, theta oscillations might be the dynamic link between hippocampal and neocortical areas (Guderian and Duzel, 2005). Consistent with the hippocampus’s key role in spatial memory processing, several studies have demonstrated links between interregional theta coupling and performance on a variety of spatial memory tasks (Colgin, 2013).

### Functional Connectivity Destruction in AD

In our study the most prominent functional connectivity differences between AD and controls were found in the delta, theta, and alpha bands in the encoding session and delta and theta bands in the retrieval session. The findings in spectral power and synchronization values seem not to be closely related to each other. Differences in synchronization between the controls and AD patients were found to be more conspicuous than those seen for the spectral measures. This is not surprising, since spectral power and synchronization attributes reflect different aspects of the EEG, and they are mathematically independent. As the underlying neurophysiological mechanisms of the findings related to the differences between the controls and AD patients are not clear, the two types of analyses may reflect different, perhaps complementary pathophysiological aspects (Hidasi et al., 2007). Distributed networks of brain regions, which are directly connected by anatomical tracts or by functional associations and with a time-varying dynamic and hierarchy, were engaged in the human brain functions (Vecchio et al., 2014). As multimodal information processing at the level of cortico-cortical projections are affected in AD, a hypothetical model of “disconnection syndrome” for AD symptomatology was suggested (Delbeuck et al., 2003). Abnormalities in functional connection between brain cortical regions were found in AD in studies using EEG, functional magnetic resonance image (fMRI), Positron emission tomography (PET), magnetoencephalographic (MEG) (Stam et al., 2006; Liu et al., 2014; Vecchio et al., 2014; Dai et al., 2015; Engels et al., 2015; Romero-Garcia et al., 2016).

The most prominent impairment in brain networks of AD were found in long distance connections, and the degrees of impairment were related to cognitive decline (Dai et al., 2015). However, these studies focused on the resting state of the brain network, which is different from the brain network associated with cognitive tasks. Our study investigated the brain networks associated with object-location memory and obviously, our results showed that the more differences of functional connections between two groups were also long distance connections which were presented in **Figures 3, 4**. This result maybe indicated that AD patients used compensational long distance network to complete object-location memory when their long-distance connections were destructed. The increased inter-hemispheric functional connectivity related to memory networks were also found in MCI patients, and the researchers believed that the results could reflect a compensatory mechanism (Bajo et al., 2010). The alterations of inter-hemispheric connections might result from axonal degeneration in anterior and posterior regions of the corpus callosum in AD patients (Di Paola et al., 2010; Wang et al., 2014; Romero-Garcia et al., 2016). Moreover, the increased functional connectivity association with visual sensory and cognitive stimulation, which reflected by the increased coherence values in gamma band, were found in AD patients compared to healthy elders (Basar et al., 2017). On the other hand, some previous studies showed decrease of coherence values in AD patients compared to healthy controls during cognition task (Guntekin et al., 2008; Basar et al., 2010; Yener and Basar, 2013). These previous literatures are highly controversial. The discrepancies could be due to differences of recoding state, recoding techniques, and analysis methods (Basar et al., 2017).

Since methods of functional connectivity were more conspicuous to changes in electrophysiological characteristics of interneuronal connectivity than spectral power (Stevens et al., 2001; Adler et al., 2003; Pogarell et al., 2005), functional connectivity may become a sensitivity biomarker for early detection of AD and contribute to finding the mechanism of nerve injury.

### Limitations

Because the number of participants was relatively low, one of our limitations is that only preliminary conclusions can be drawn. The difficulty of performing a given task may limit the number of individuals involved in such research. These difficulties may be the reason why there is no simple and commonly recognized approach to assess the spatial memory of AD patients. As a result, we only analysis the item location in left and successful recall subsequently, the right/left location was primitive one of spatial context. And the participant subjects were merely mild AD patients. In order to facilitate operator response, we only use two buttons for each subject. However, this paradigm may lead to subjects using a different memory strategy than subjects using three buttons.

## Conclusion

Our results indicate that the function of object-location memory in AD patients is significantly impaired when compared to healthy elders. In the AD group, there was higher spectral power in the alpha band frequency during memory encoding, and the theta and alpha bands during memory retrieval. Strengths of connectivity were clearly increased in the AD group when compared to the healthy control group prominent in theta, alpha, and beta band frequencies in memory encoding and theta band frequency in memory retrieval. Our findings provide support for the hypothesis that AD patients may use compensation mechanism to memory items successfully.

## Author Contributions

YH, JJ, and WW conceived the study and coordinated the experiments. YH and KW performed the experiments and analyzed the data. YH wrote the manuscript. JJ and WW revised the manuscript. All authors read and approved the final manuscript.

## Conflict of Interest Statement

The authors declare that the research was conducted in the absence of any commercial or financial relationships that could be construed as a potential conflict of interest.

## Footnotes

1 https://sccn.ucsd.edu/eeglab/#
